# Classification of skin lesions using transfer learning and augmentation with Alex-net

**DOI:** 10.1371/journal.pone.0217293

**Published:** 2019-05-21

**Authors:** Khalid M. Hosny, Mohamed A. Kassem, Mohamed M. Foaud

**Affiliations:** 1 Department of Information Technology, Faculty of Computers and Informatics, Zagazig University, Zagazig, Egypt; 2 Department of Information Systems, Modern Academy, Cairo, Egypt; 3 Department of Electronics and Communication, Faculty of Engineering, Zagazig University, Zagazig, Egypt; Newcastle University, UNITED KINGDOM

## Abstract

Skin cancer is one of most deadly diseases in humans. According to the high similarity between melanoma and nevus lesions, physicians take much more time to investigate these lesions. The automated classification of skin lesions will save effort, time and human life. The purpose of this paper is to present an automatic skin lesions classification system with higher classification rate using the theory of transfer learning and the pre-trained deep neural network. The transfer learning has been applied to the Alex-net in different ways, including fine-tuning the weights of the architecture, replacing the classification layer with a softmax layer that works with two or three kinds of skin lesions, and augmenting dataset by fixed and random rotation angles. The new softmax layer has the ability to classify the segmented color image lesions into melanoma and nevus or into melanoma, seborrheic keratosis, and nevus. The three well-known datasets, MED-NODE, Derm (IS & Quest) and ISIC, are used in testing and verifying the proposed method. The proposed DCNN weights have been fine-tuned using the training and testing dataset from ISIC in addition to 10-fold cross validation for MED-NODE and DermIS—DermQuest. The accuracy, sensitivity, specificity, and precision measures are used to evaluate the performance of the proposed method and the existing methods. For the datasets, MED-NODE, Derm (IS & Quest) and ISIC, the proposed method has achieved accuracy percentages of 96.86%, 97.70%, and 95.91% respectively. The performance of the proposed method has outperformed the performance of the existing classification methods of skin cancer.

## Introduction

Skin cancer is one of the most-deadly kinds of cancers [1]. Essentially, melanoma and non-melanoma are the most known skin cancer types [2]. Death rate and incidence have increased significantly in last years because of melanoma lesions. The rate of curing can reach over 90% where physicians would save patients’ life if these lesions were detected in early stage [3]. Commonly, visual examination of skin cancer is difficult and may lead to wrong detection of lesions because there is a high similarity between different types of skin lesions (melanoma and non-melanoma) [4]. Therefore, the automatic classification of skin lesion images by using the image processing techniques and artificial intelligence is a successful alternative solution of the visual examination [5].

About 75% of deaths related to skin cancer come from Melanoma lesions [6]. Survival rate of patients could be increased if melanoma was recognized accurately in its early stages [7]. Manual detection of melanoma requires well-trained specialists to overcome variations of inter-observation. Thus, if the melanoma recognition has been done automatically, it will increase efficiency and accuracy of the early detection of this kind of cancer.

The performance of melanoma diagnosis has been improved using the dermoscopy technique [8]. Dermoscopy is a noninvasive imaging technique for skin, which is able to capture illuminated and magnified images of skin lesion to increase the clarity of the spots. The visual effect of the deeper skin level can be enhanced if the skin surface reflection is removed [9]. However, automatic recognition of melanoma dermoscopy images is a challenging task due to few factors. First, segmentation of skin lesions is a very difficult and tedious task because of lesions variation of intraclass such as, texture, size, color, shape and location. Second, the high similarity between melanoma and non-melanoma lesions. Finally, the surrounding environmental conditions like hair, veins, and charts of color calibration and ruler marks.

There are many trials to overcome these challenging problems. Early, researchers tried to distinguish non-melanoma and melanoma lesions using low-level hand-crafted features [10]. Other researchers presented algorithms to select the proper hand-crafted features but features suffer from high visual similarity, huge variations of intraclass and artifacts of dermoscopy images which lead to bad results [11]. On the other hand, another group of researchers applied segmentation methods to discard background and unneeded features [12]. In fact, the procedures of segmentation and classification are based on low-level features with low discrimination capabilities which led to bad results [13].

A set of high-level intuitive features (HLIF) have been proposed by Amelard et al. [14] to describe the amount of lesion border irregularity. To get more semantic meaning of the feature set, a small set of HILF for low-level feature was used. This allowed the proposed system to provide an intuitive rationale decision for the classification. Their system achieved a classification rate of 87.38%. Karabulut and Ibrikci [15] utilized the convolutional neural network and support vector machine (SVM) to classify the skin cancer. Preprocessing steps have been used for enhancement and segmentation where the local binary pattern (LBP) and the difference of block texture analysis of inverse probabilities were applied. Their system achieved an accuracy rate of 71.4%.

Almaraz-Damian et al. [16] proposed a computer-aided diagnosis system in which the features were extracted using the ABCD rule, and the SVM was used for classification. The accuracy of their system was 75.1%. Giotis et al. [17] used the descriptors of color and texture to extract the region of lesion for classification purposes. Their system achieved a classification rate of 81%. Jafari et al. [18] reduced the noise in the input images by a guided filtering method, and then they applied the ABCD rule to extract the skin lesions features. The accuracy of their method was 79%.

The deep convolutional networks (DCNN) has the ability to learn from features hierarchically. These networks have been applied in analyzing the medical images [19]. The level of image classification accuracy has been increased by using the DCNN [20] especially with large datasets [21]. Since the implementation of the DCNN required big number of images to achieve high classification rates, insufficient number of skin cancer colorful images represents an additional challenge in detection of the skin lesions.

Nasr-Esfahan et al. [22] applied the deep learning with clinical images. They used a correlation for the illumination, and then they segmented the skin lesion in a preprocessing step to increase the accuracy of their system. The enhanced and segmented images were sent to the CNN for feature extraction and classification. The accuracy of this system was 81%. For Plain photography, a computer-based analysis was proposed by Kostopoulos et al. [23]. The features were extracted by Probabilistic Neural Network (PNN) to decide the type of skin lesion. Their system achieved a classification rate equal to 76.2%.

Premaladha and Ravichandran [24] presented a computer-aided diagnosis system (CAD) for skin lesion classification by combining the supervised algorithms and deep learning. The input images were enhanced using the contrast limited adaptive histogram equalization technique (CLAHE), and then the normal skin was separated by the median filter with the Normalized Otsu’s Segmentation (NOS). They utilized the Artificial Neural Network (ANN) and Deep Learning Neural Network (DLNN) where the achieved classification rates were 90.12% and 92.89% respectively.

Pham et.al. [25] used data augmentation and deep CNN to improve classification performance of melanoma. They used Inception V4 architecture based on GoogleNet and achieved a classification rate equal to 89%. Esteva et al. [26] used a single trained end-to-end CNN to classify skin lesions. They classified three classes called melanomas, seborrheic keratosis and benign/nevus. They used the Inception v3 pertained architecture from google and achieved moderately low classification rate of 72.1%. Yu et al. [27] presented an automated method for melanoma recognition in dermoscopy images using very deep residual networks. They used the residual learning to deal with overfitting and degradation problems. They built a Fully Convolutional Residual Network (FCRN) for classification. The results of experiments show a classification rate of 85.5%.

Accurate detection of melanoma and high classification rates are very essential in the early detection of skin cancer. The general classification rates achieved by the existing methods are less than 90%, which is unacceptable. Achieving high classification rate is essential in CAD systems for skin cancer. This motivates the authors to present a new deep learning neural network-based method, which achieves a classification rate of 97%.

In this paper, a pre-trained deep convolutional neural network system is used for automated skin lesion classification. The last fully connected layer has been dropped out and replaced by a softmax with random weights to be appropriate for the task of classification in this paper. The contributions of the proposed system can be summarized as follows:

1. We utilized Alex-Net which outperformed the other deep architectures based on the following attractive characteristics:
  • Each layer has much more filters.
  • In addition to the stacked convolutional layers, each convolution layer is followed by a pooling layer.
  • Instead of Tanh, logistic, arctan or Sigmoid as activation function it uses RelU function which reduce likelihood of vanishing gradient and it is more biological inspired.
  • Its training is 5 times faster comparing with others deeper architecture speed.
  • Alex-Net does not require a specific hardware. It could work-well with limited GPU which is an additional characteristic.
  These characteristics motivated the authors to utilize a modified Alex-Net in skin lesions classification. The proposed method outperformed the existing deep learning-based skin lesions classification methods in terms of the classification rate, the sensitivity and the specificity.
2. Second, significantly improved the classification rate of skin lesions which outperformed the rates obtained by existing similar methods.
3. Third, since we work for binary and multi-class classification, the softmax works well. The output of using softmax is the probabilities range, from 0 to 1 while the summation of all the probabilities will be equal to one. In multi-class classification model, each class probability is returned but the high probability will be to the target class. Based on this characteristic and its simplicity, we replaced the classification layer of the Alex-Net by the softmax.
4. Finally, Utilization of random and fixed rotation augmentation approaches increased the number of skin images which enabled a well-training of the Alex-Net and led to the highly classification rate.

The rest of this work is organized as follows: A brief description of the utilized DCNN is presented in section 2. The proposed method for color skin images classification is described in section 3. Description of the performed experiments and the obtained results are presented in section 4. A discussion for the proposed method and literature methods are discussed in section 5. The conclusion is presented in section 6.

## Background

DCNN consist of neural networks, which have a number of convolutional layers to extract features from images and classify these images [28]. The difference between the original data used to train DCNN and the data used for testing will be minimized in the training phase with different scale or size but with the same feature. The feature can be extracted and classified using deep network well [29]. So DCNN can be used in the task of skin lesion detection and classification. The reasons behind that is noise, aberrations, and artefacts in addition to limitation of labeled images. Another reason is that dermoscopic images may have large variation for same features plus the visual similarity of different type of lesions. So, a large dataset must be used with DCNN for training to overcome these challenge [30,31].

CNN has been used to improve the performance in many applications like natural language processing [32]. There are many DCNN architecture like LeNet, AlexNet, ZFNet, GoogLeNet, and VGGNet [33–34] that are available to be used in many different applications. In this work, AlexNet has been used and evaluated.

### AlexNet CNN model

Krizhevsky et al. [35] has developed AlexNet to use ImageNet Large-Scale Visual Recognition Challenge (ILSVRC) [36]. The first layer of AlexNet is used to filter the input image. The input image must have width (*W*), height (*H*), and depth (D); 227×227×3 in which *D* = 3 refers to red, green, and blue. As mentioned above, the first convolutional layer used to filter the input color image which has a number of kernels (*K*) equals to 96 with a filter (*F*) of size 11x11 in addition to 4 pixels is called stride (s). The distance between responsive field centers of neighboring neurons in the kernel map is called stride. The mathematical formula, ((*W*−*F*+2*P*)/*S*)+1, is used to compute the output size of the convolution layer where P refers to number of padded pixels, which equals here to zero. By using this formula, the volume size of the convolution layer output is ((227−11+0)/4)+1 = 55. The second convolutional layer input will be (55×55×*no of filters*) and the number of filters in this layer is 256. Since the work of this layer is distributed over 2 GPUs, the load for each one is divided by 2 for the two GPUs.

The following is the convolutional layer followed by the pooling layer. The pooling layer tries to reduce the dimensionality of each feature map while keeping important features, where pooling may be Sum, Max, Average, etc. AlexNet uses a max pooling layer. The input of this layer is 256 filters. Each filter is 5×5×256 in addition to 2 pixels as a stride. By using two GPUs, the work will be divided to 55/2×55/2×256/2≈27×27×128 for each GPU.

**Fig 1:**
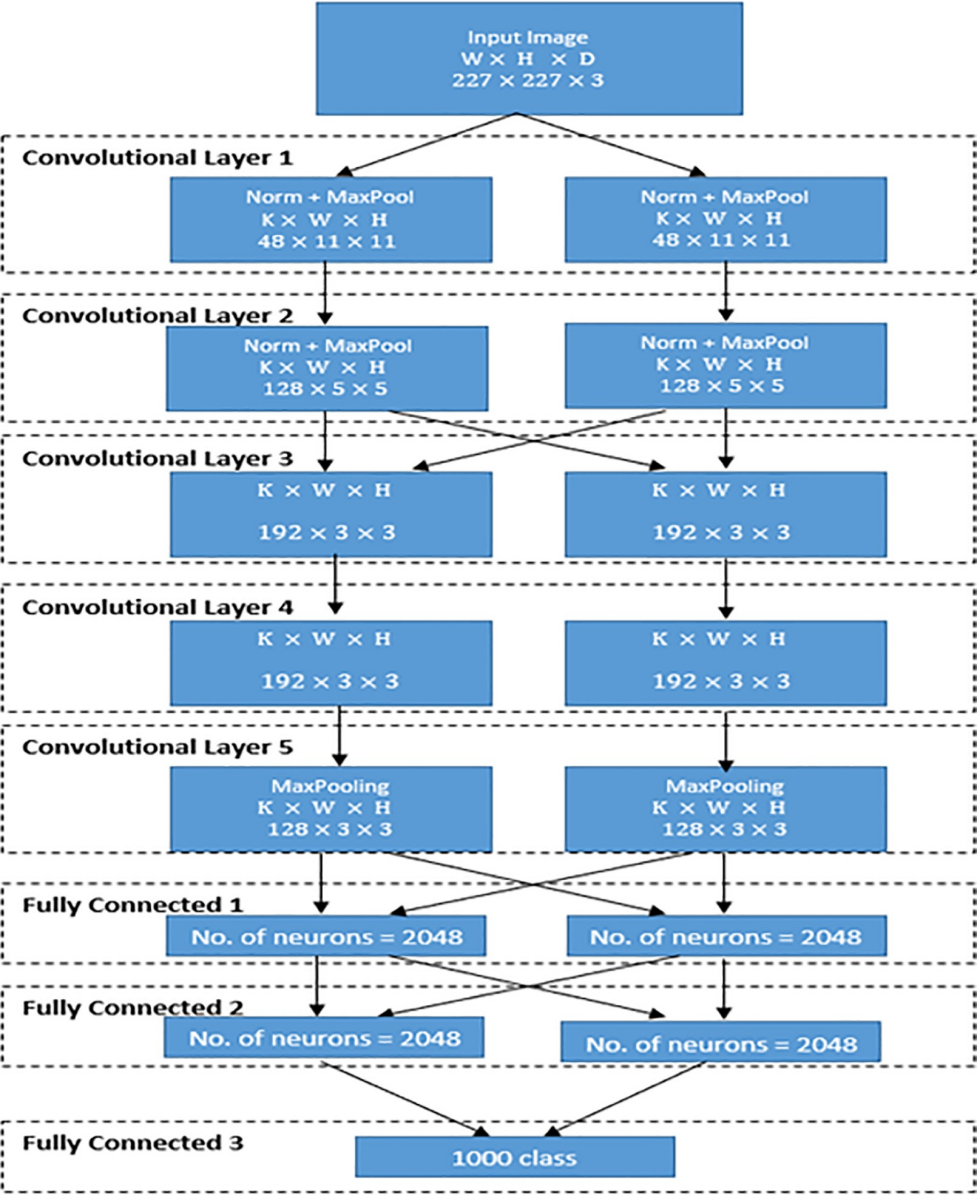
Architecture of Alex-Net: W refers to width, H refers to height, D refers to depth, and K refers to number of filters.

The pooled and normalized output of the second convolutional layer is connected to third layer with 384 kernels, each of size 3×3. There are 384 kernels of size 3×3 for the fourth convolutional layer and they will be divided over 2 GPU so each GPU load will be 3×3×192. fifth convolutional layer have 256 kernels each kernel of size 3×3 and they will be divided over 2 GPU so each GPU load will be 3×3×128. It must be noted that the third, fourth and fifth convolutional layers are created without any normalization and pooling layers. The output of these three convolutional layers are passed as input to number of 2 fully connected layers where each layer contains 4096 neurons. Fig 1 illustrates the overall architecture of Alex-Net to classify different classes using imageNet [36] as a training dataset.

## The proposed method

In this section, the proposed method to classify the colored images for skin lesions is described. This section is divided into two subsections. The augmentation process for the colored images is presented in the first subsection. The process of transfer learning which is applied to the deep network is described in the second subsection.

### The augmentation process

To gain good performance with DCNN, a huge number of training images are needed. Generally, the small number of labeled medical images is a big challenge. The available datasets of color skin images, such as 2017 ISIC Challenge data set [37], MED-NODE [17], Dermatology information system [38] and DermQuest [39] contain small numbers of labeled images where ISIC dataset consists of 2000 divided to 374, 254, and 1372 samples for Melanoma, Seborrheic Keratosis, and Nevus respectively.

The second dataset, MED-NODE, contains 170 images divided to 70 and 100 images for melanoma and nevus images respectively. This dataset comes from the digital image archive of the department of Dermatology, University Medical Center Groningen (UMCG) in Netherlands. It is used for the development and testing of the system for skin cancer detection from macroscopic images.

The third one has collected from the publicly databases by [14] which is available online from Dermatology Information System [38], and The DermQuest [39]. Third dataset consists of 206 images of skin lesion divided to 119 and 87 images for melanoma and nevus. These images are obtained by using standard consumer-grade cameras in varying and unconstrained environmental conditions.

In order to increase the number of trained images, we have followed some of the augmentation techniques that have been discussed in [40]. the labelled data melanoma, seborrheic keratosis and nevus images are rotated with different rotation angles in two ways. The first way is the random rotation, while the second one is the rotation with fixed step angle equal to 5^0^. Each image rotated 72 times with random different angels in the range from 0^0^ to 355^0^. The same number of rotations,72, was carried out with rotation angles 0^0^ to 355^0^ with a fixed step 5^0^. The number of images in the first dataset becomes (355/5+1)×374 = 26928 melanoma images, (355/5+1)×254 = 18288 Seborrheic Keratosis images, and (355/5+1)×1372 = 98784 nevus images. Similarly, the second and the third dataset will be (355/5+1)×70 = 5040, (355/5+1)×119 = 8568 melanoma images and (355/5+1)×100 = 7200, (355/5+1)×87 = 6264 nevus images respectively. The augmentation process is done only to increase the number of the images in the dataset without any medical reason.

### Transfer learning

Despite the large increase in the number of trained images, the available size of the dataset is insufficient to train a new deep model from scratch. To overcome this problem, the theory of transfer learning is applied to the pre-trained AlexNet architecture in three different ways. First, the classification layer is replaced to softmax layer with two or three classes. Second, the weights have been fine-tuned and the back-propagation is run to train the new weights. A small learning rate is used where the weights of the convolutional layer are not changed dramatically, while weights of the fully connected layers are randomly initialized. The stochastic gradient descent (SGD) algorithm used to update the weights on the network is based on the used datasets of skin cancer. Finally, the datasets are augmented to increase the number of images that are available to train the deep network. This process resulted in the optimal weights and achieved a good classification rate with the new replaced softmax layer.

As mentioned above, melanomas have huge intraclass variation, and there is a high degree of visual similarity between melanoma and non-melanoma lesions, which severely influences the recognition performance especially when performing skin lesion classification using the original limited dermoscopy images.

## Experiments and results

**Fig 2:**
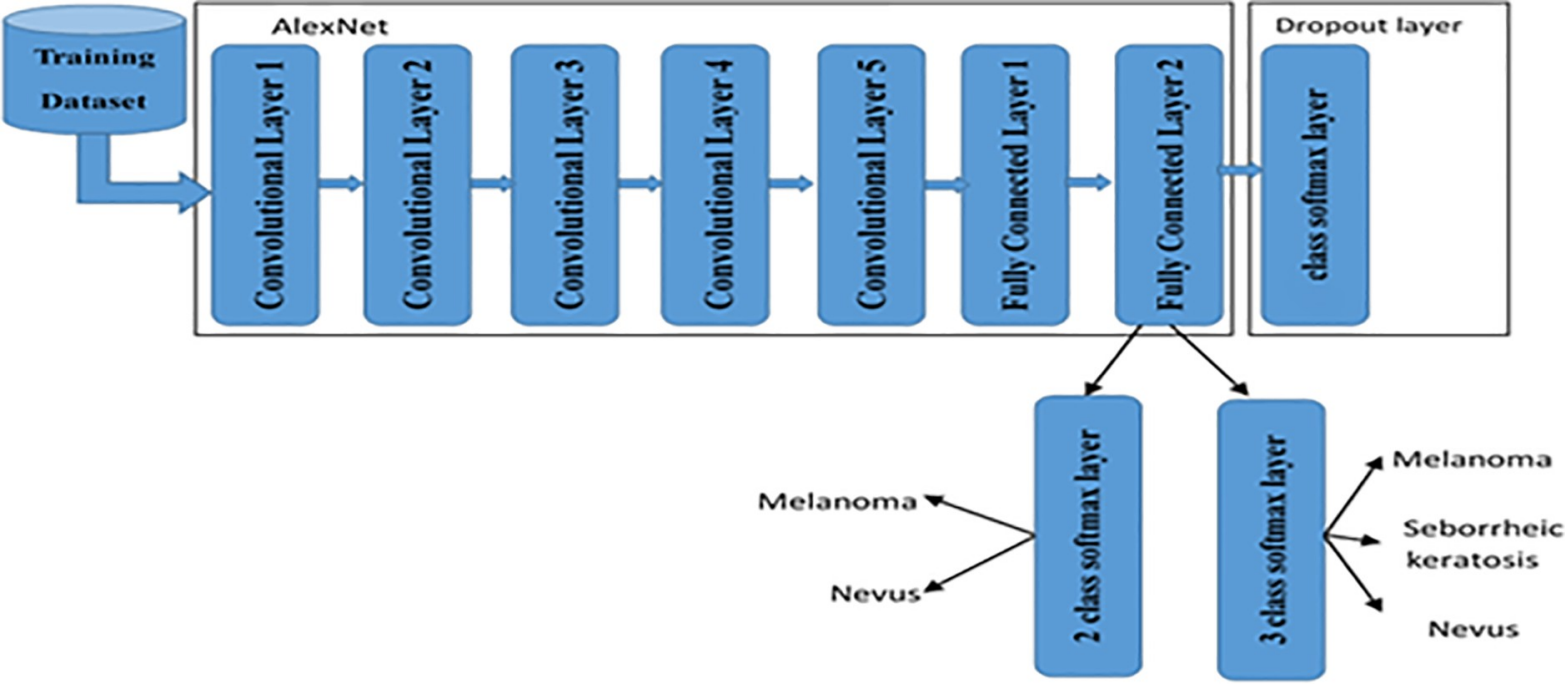
Modified Alex-Net.

Experiments are performed using an IBM computer equipped with a core i5 processor, 8 GB DDRAM and a NVIDIA GeForce 920M graphic card. The MATLAB 2017 x64-bit is used to execute the coded program. Three datasets, ISIC, MED-NODE, and DermIS—DermQuest, of RGB colored skin images are used in these experiments. The first dataset consists of three labeled data/classes, melanoma, seborrheic keratosis, and nevus. The second and the third datasets consist of only two labeled data/classes, melanoma and nevus. The code is converted from MATLAB 2017 to CUDA to be run over GPU. Using GPU enables us to use a huge number of training data with low error rate of models. In many works, like that with DCNN, the classification layer may be dropped out and replaced with other classification methods like multi-class SVM. In this work, the classification layer called softmax is replaced with a new softmax layer to be appropriate for skin lesion where three classes are used. Fig 2 illustrates the modified pre-trained AlexNet with the new softmax layer.

There are two kinds of performed experiments with the three datasets. The first one is to evaluate the proposed method using original datasets without image augmentation. The second one is to evaluate the proposed method with augmented datasets. All experiments are performed with fixed values of the batch size, 10, the number of training epochs, 32, and the initial learning rate, 0.001. All color images are segmented using the segmentation methodology [41].

Four evaluation measures are used to evaluate the performance of proposed method. These measures are accuracy, sensitivity, specificity, and precision [42]. These measures are computed using the following equations:

$$Accuracy=\frac{t_{p}+t_{n}}{t_{p}+f_{p}+f_{n}+t_{n}}$$

$$Sensitivity(TPR)=\frac{t_{p}}{t_{p}+f_{n}}$$

$$Specificity(TNR)=\frac{t_{n}}{f_{p}+t_{n}}$$

$$Precision(PPV)=\frac{t_{p}}{t_{p}+f_{p}}$$

Where *t*_*p*_, *f*_*p*_, *f*_*n*_, *and t*_*n*_ refer to true positive, false positive, false negative, and true negative respectively. The acronyms, TPR, TNR, and PPV refer to true positive rate, true negative rate, and positive prediction value. The rates of true negative and false positive should be large and small, which makes most of the points fall in the left part of the receiver operating characteristic (ROC) curve [43].

All experiments start by loading the color images form the data source, then by passing it to the segmentation step. According to the pre-trained AlexNet, the size of the input image cannot exceed 227×227, and the depth-limit of the image is 3. Therefore, after segmentation, a check step is performed to ensure the suitability of the image’s size. If the size of the image exceeds the size limit, a resizing process to 227×227×3 for width, height, and depth is imperative.

In the first type of experiment, the 10-fold cross validation have been used to divided MEDNODE and DermIs–DermQuest dataset into groups for training and testing without any augmentation. Each group have been used at least once as training and once as testing but not in the same run. Then the modified AlexNet after applying transfer learning theory have been used. This process was repeated 10 time and the average accuracy for the 10 runs times was computed to be the overall accuracy of the proposed model. In the first type of experiment, runs are performed using the original datasets of color images without any augmentation. The is applied where the is pre-trained. The classification layer, softmax, is modified to work with 2 classes instead of the ImageNet classes.

**Fig 3:**
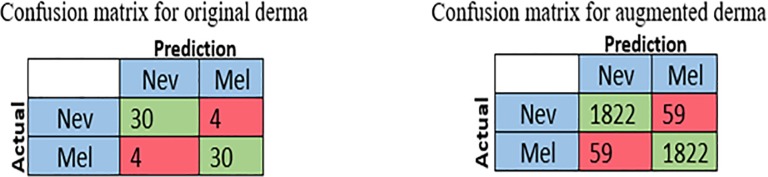
Confusion matrix for original and augmented derma dataset.

The first run was done with the DermIS—DermQuest dataset which contains low quality images of two classes, melanoma and nevus. To evaluate the performance of the proposed method, the values of the four measures are computed for each class separately. Therefore, the average value for these measures are computed. The average of the computed measures is 88.24%, 86.79%, 86.79%, and 89.01% for accuracy, sensitivity, specificity, and precision respectively. The confusion matrix of this experiment is shown in Fig 3.

**Fig 4:**
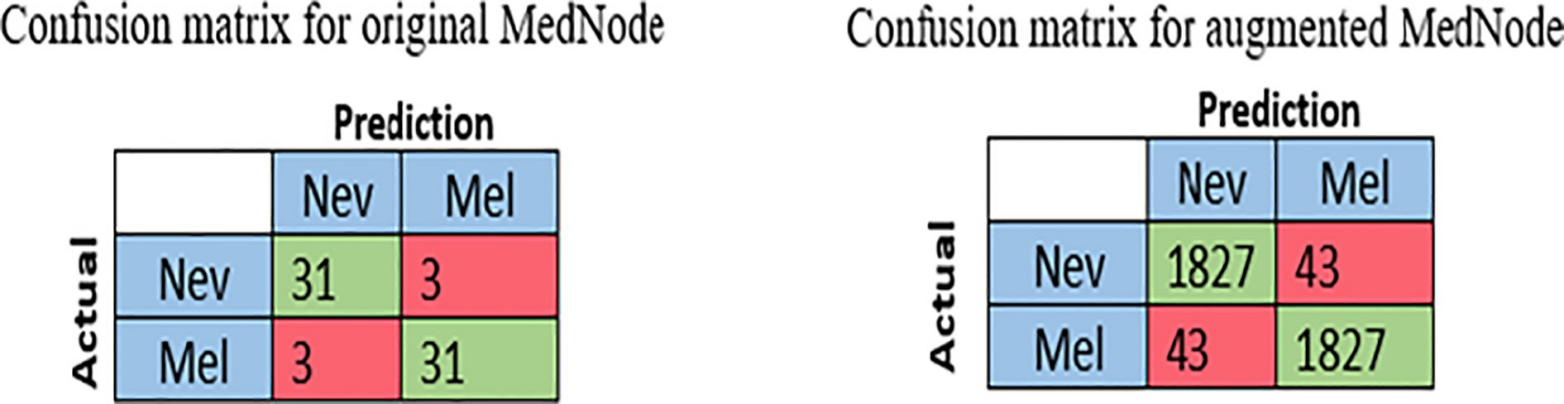
Confusion matrix for original and augmented Mednod dataset.

The second run is performed using the same conditions and architecture with the second dataset, MED-NODE. The MED-NODE dataset consists of high quality dermoscopy images. This dataset is divided into two classes, melanoma and nevus. The pre-trained AlexNet that has transferred learning with modified softmax layer to be appropriate with two classes of skin lesions. The average values of measures are computed where these values are 91.18% for average accuracy of the two classes, 91.43% for average accuracy of sensitivity and specificity, and 90.70% for average precision. The confusion matrix of this experiment is shown in Fig 4.

**Fig 5:**
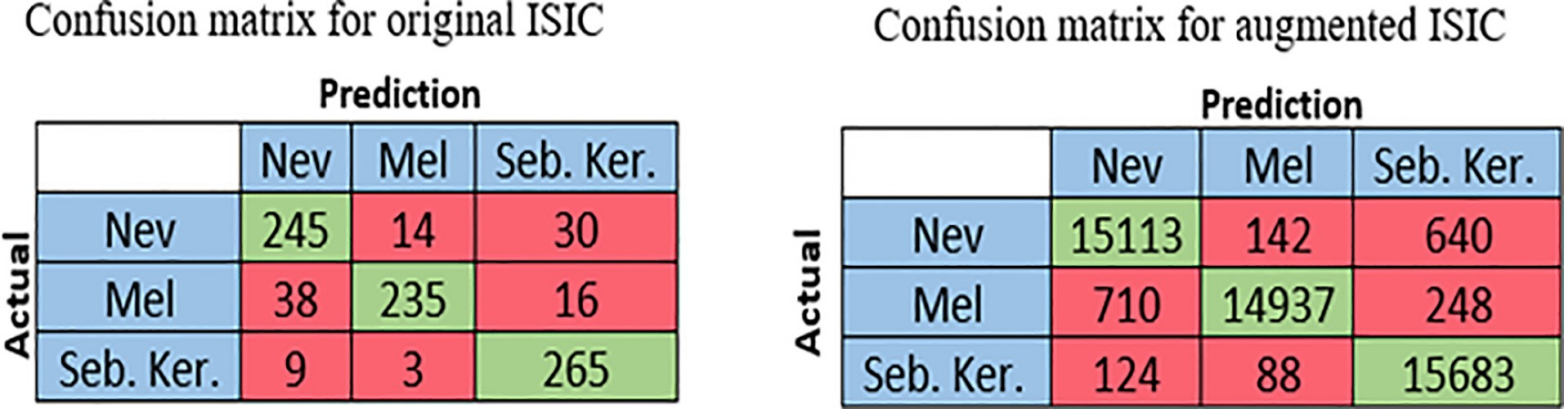
Confusion matrix for original and augmented ISIC dataset.

The ISIC dataset is used in the third run where this dataset consists of three classes, melanoma, seborrheic keratosis, and nevus. The ISIC dataset is relatively big and originally divided into training and test groups; so, we ignore 10-fold cross validation. Similarly, the transfer learning is applied to the pre-trained AlexNet where the softmax layer is modified to be worked with three classes. The values of batch size, the number of training epochs and initial learning rate were fixed for all runs as 10, 32, and 0.001 respectively. The average computed for all measures, accuracy sensitivity, specificity, and precision were 87.31%, 62.02%, 79.07%, and 73.07% respectively. The confusion matrix of this experiment is shown in Fig 5.

The second kind of experiments is performed with the same datasets, DermIS- DermQuest, MED-NODE, and ISIC. DermIS _dermQuest, and MED-MODE dataset have been splitting into training and testing groups using 10-fold cross validation. These groups in addition to the ISIC training and testing dataset groups are augmented by rotating each image with 55 different rotation angles ranging from 0^0^ to 355^0^ with a constant step 5^0^.

The DermIS- DermQuest is used in the first run of this experiment. The original colored images of this datasets are segmented to reduce the size and remove unwanted complicated background. The segmentation step is performed before the augmentation process, which is applied to all segmented color images. As a result of augmentation, the number of color images becomes 4620 and 4785 for melanoma and nevus respectively. A size-check constraint, 227×227×3, is applied to all input images as done in the first experiment. The same values of batch size,10, the number of training epochs, 32, and initial learning rate, 0.001 are applied. The classification layer softmax is modified to two classes, melanoma and nevus. The same four measures are used to evaluate the performance of the modified pre-trained AlexNet. The computed average values of these measures are 96.86% for accuracy, 96.90% for sensitivity and specificity and 96.92% for precision. The confusion matrix of this experiment is shown in Fig 3.

The second run is performed with the MED-NODE dataset. Like previous runs, the original color images are segmented and then the augmentation process is performed where the number of images becomes 3850 and 5500 for melanoma and nevus respectively. The size of all images is determined where images with exceeded size are resized to 227×227×3 for width, height, and depth. The performance measures are computed where the average values of these measures are 97.70%, 97.34%, 97.34%, and 97.93% for accuracy, sensitivity, specificity, and precision respectively. The confusion matrix of this experiment is shown in Fig 4.

The third experiment is performed with the ISIC dataset. This dataset consists of three classes, melanoma, seborrheic keratosis, and nevus. Therefore, the modified softmax layer will be modified to work with these three classes. After segmentation process, the dataset is augmented where the number of images becomes 20570, 13970 and 75460 images for melanoma, seborrheic keratosis, and nevus respectively. The size of all input images must not exceed the size 227×227×3. The average values of the performance measures are 95.91%, 88.47%, 93.00%, and 92.34% for accuracy, sensitivity, specificity, precision, and negative predication value respectively. The confusion matrix of this experiment is shown in Fig 5.

Table 1 gives an overview of the obtained results for the performed experiments. It is clear that the augmentation processes significantly improve the classification rates. The proposed method achieved a very high classification rates with different datasets.

**Table 1:** The proposed model accuracy.

Dataset	Original dataset				Augmented dataset			
	Average accuracy	Average sensitivity	Average specificity	Average Precision	Average accuracy	Average sensitivity	Average specificity	Average Precision
**DermIS-** **DermQuest**	88.24	86.79	86.79	89.01	96.86	96.90	96.90	96.92
**MED-NODE**	91.18	91.43	91.43	90.70	97.70	97.34	97.34	97.93
**ISIC**	87.31	62.02	79.07	73.07	95.91	88.47	93.00	92.34

## Discussion

**Fig 6:**
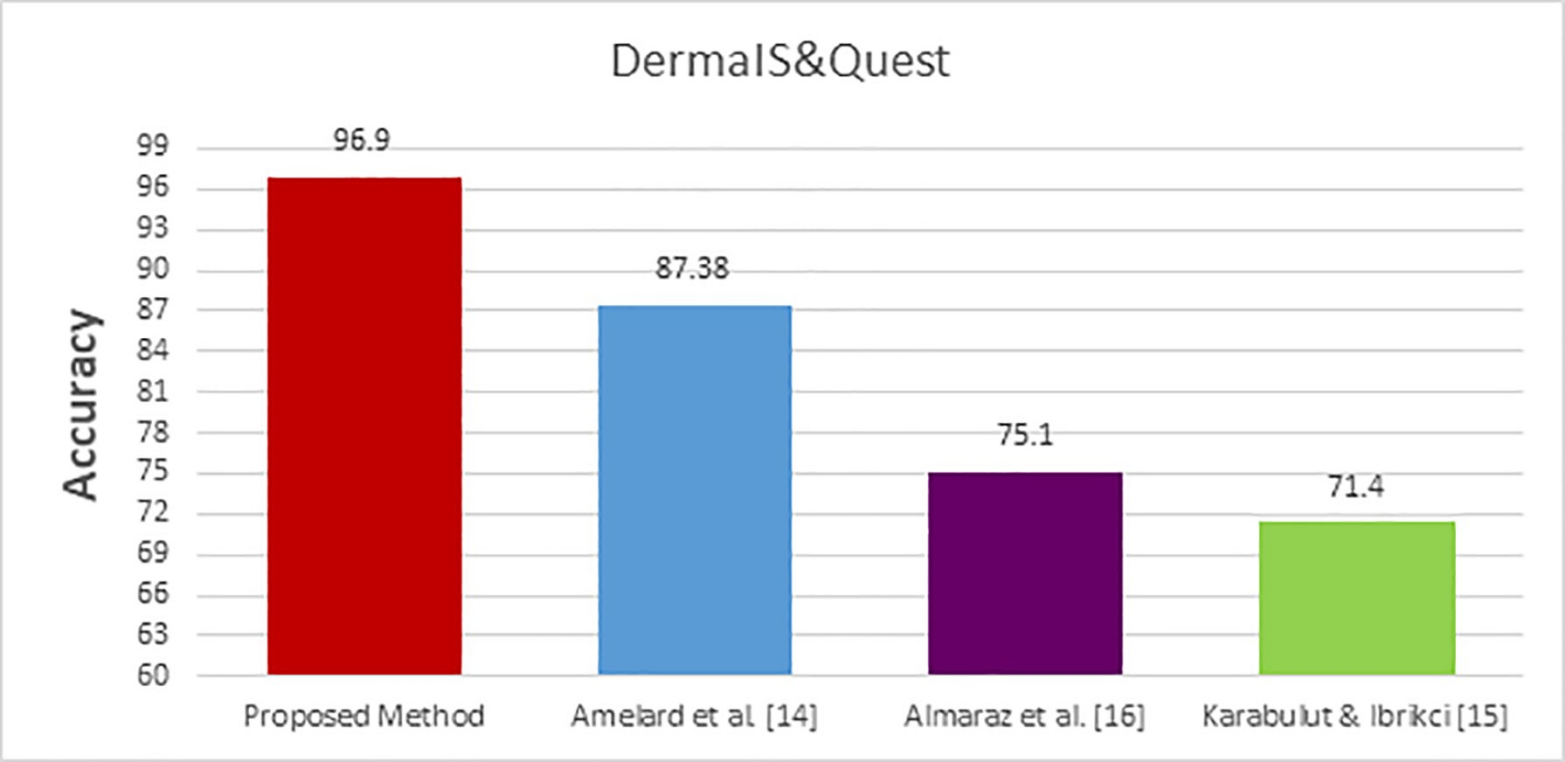
Comparative study bars for DermIS- DermQuest.

**Fig 7:**
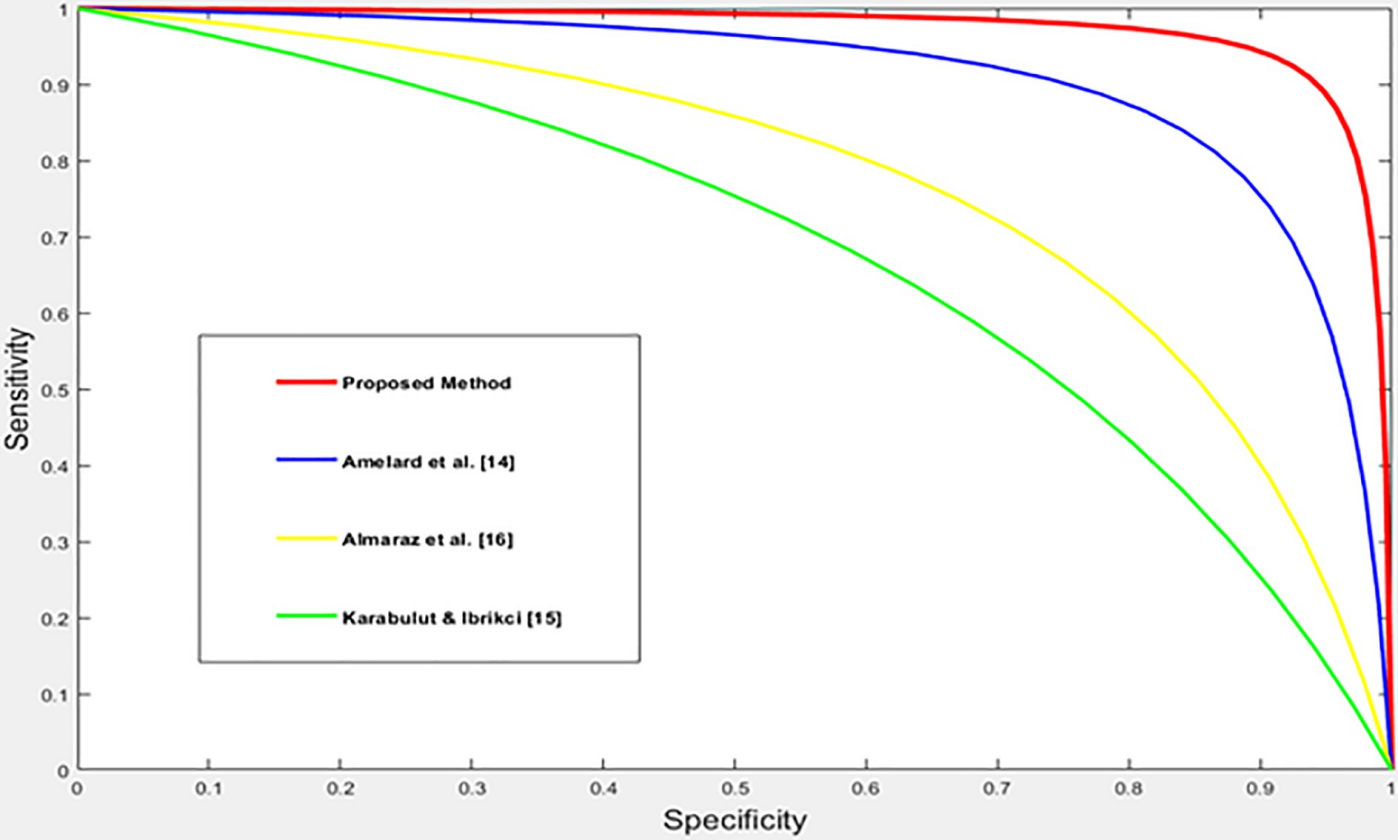
The ROC curves for the different methods with the DermIS- DermQuest dataset.

The performance of the proposed method is compared with the performance of the existing skin cancer classification methods [14–18,22–27]. The three datasets, DermIS- DermQuest, MED-NODE, and ISIC are used in this comparison. The comparative study has been done using the results as they appear in the corresponding papers. The accuracy measure and ROC curves are used as a quantative and qualitative measures to compare the performance of the different methods. The comparative study is divided into three groups based on the used dataset. In the first group, the performance of the proposed method is compared using the DermIS- DermQuest dataset. In the second group, the performance of the proposed method is compared with the performance of the existing methods [17, 18, 22, 23 and 24] using the MED-NODE dataset. The dataset, ISIC, is used in the last group where the performance of the proposed method is compared with the performance of the existing methods [25,26 and 27]. The obtained results of the first group using the DermIS- DermQuest dataset are shown in Table 2. The obtained results are visualized and displayed in Fig 6. The ROC for the proposed method and the existing classification methods [14–16] are displayed in Fig 7.

**Table 2:** DermIS- DermQuest dataset comparative study.

	Preprocessing (Enhancement)	Segmentation	Image Type	Classification Method	Accuracy (%)	Sensitivity (%)	Specificity (%)
Karabulut & Ibrikci [15]	Yes	Yes	Gray-scale	CNN	71.4	71.9	70.8
Almaraz et al. [16]	Yes	Yes	RGB	SVM	75.1	94.94	100
Amelard et al. [14]	Yes	Yes	RGB	SVM	87.38	90.76	82.76
Proposed Method	No	Yes	RGB	DCNN	96.86	96.90	96.90

**Fig 8:**
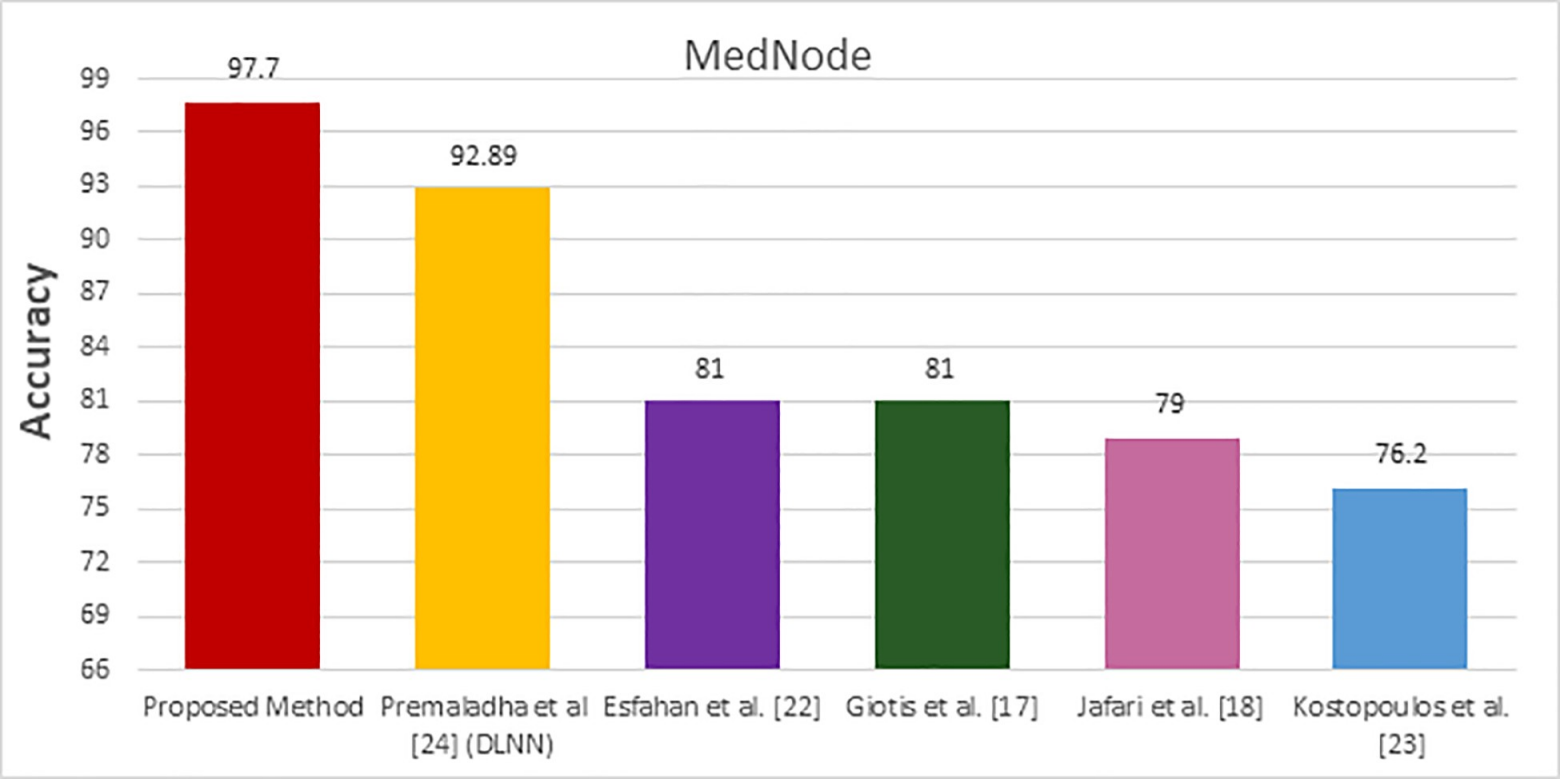
MedNode comparative study bars.

**Fig 9:**
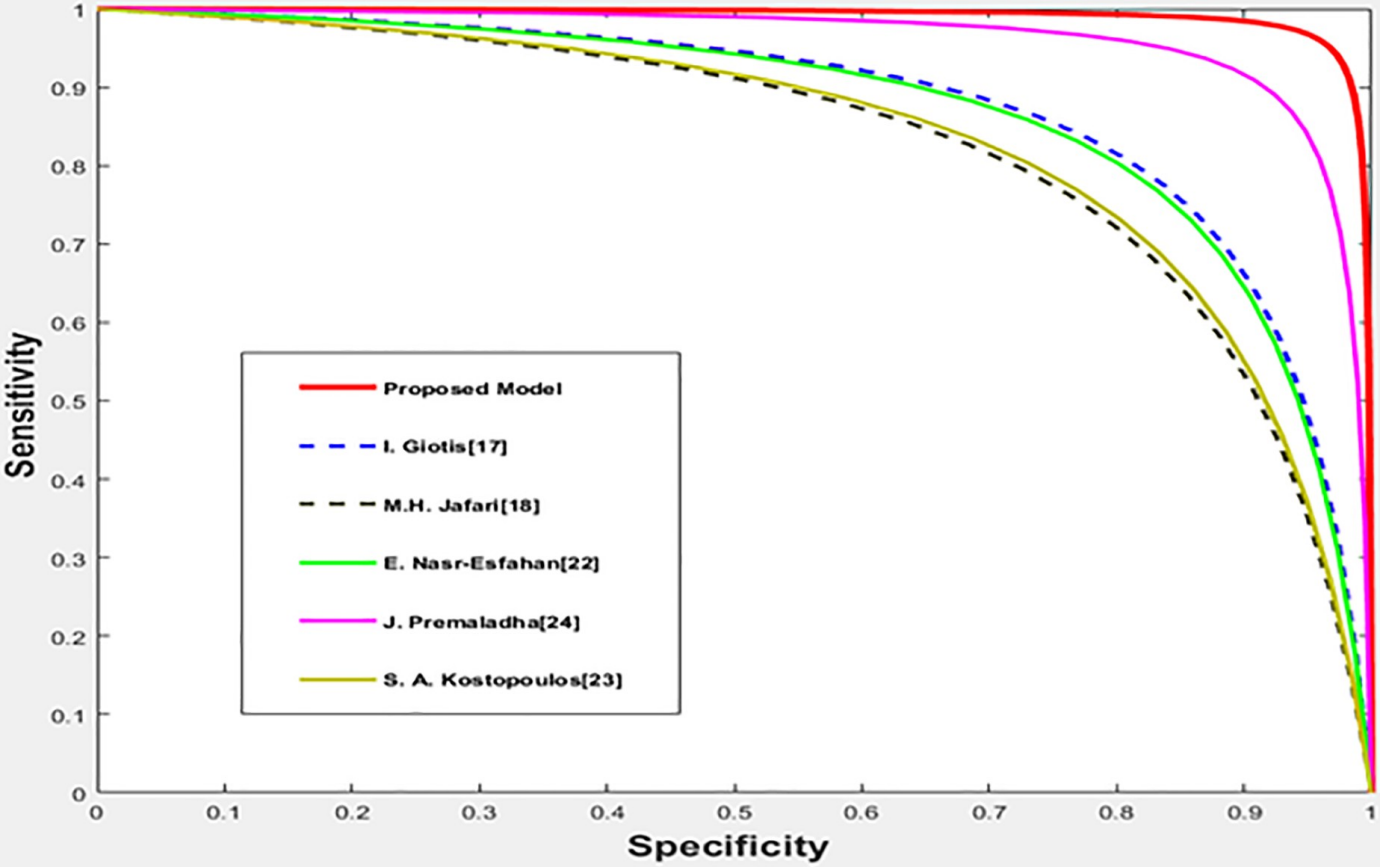
The ROC curves for the different methods with the MED-NODE dataset.

The performance of the proposed method is compared with the performance of the existing methods [17, 18, 22, 23, and 24] using the MED-NODE dataset where the obtained results are shown in Table 3. The achieved accuracies are visualized and displayed in Fig 8 while the different ROC curves are plotted in Fig 9.

**Table 3:** MED-NODE dataset comparative study.

	Preprocessing (enhancement)	Segmentation	Image Type	Classifier	Accuracy (%)	Sensitivity (%)	Specificity (%)
Kostopoulos et al. [23]	Yes	Yes	RGB	Probabilistic Neural Network	76.2	73.9	77.8
Jafari et al. [18]	Yes	Yes	RGB	SVM	79	90	72
Giotis et al. [17]	Yes	Yes	RGB	Majority vote	81	80	81
Esfahan et al. [22]	Yes	Yes	RGB	CNN	81	81	80
Premaladha, and Ravichandran [24]	Yes	Yes	Gray-scale	DLNN	92.89	94.83	90.46
Proposed Method	No	Yes	RGB	DCNN	97.70	97.34	97.34

**Fig 10:**
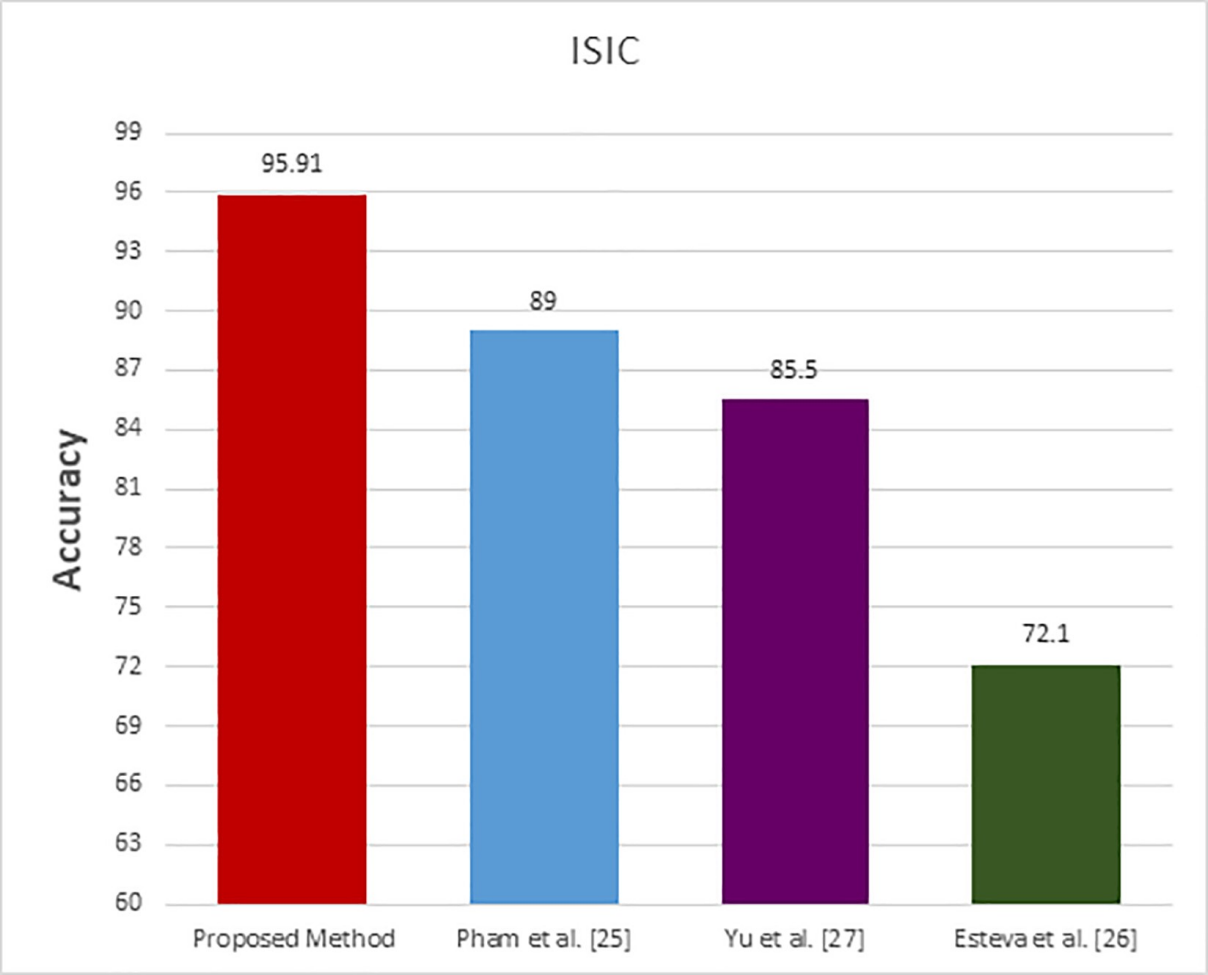
ISIC comparative study bars.

**Fig 11:**
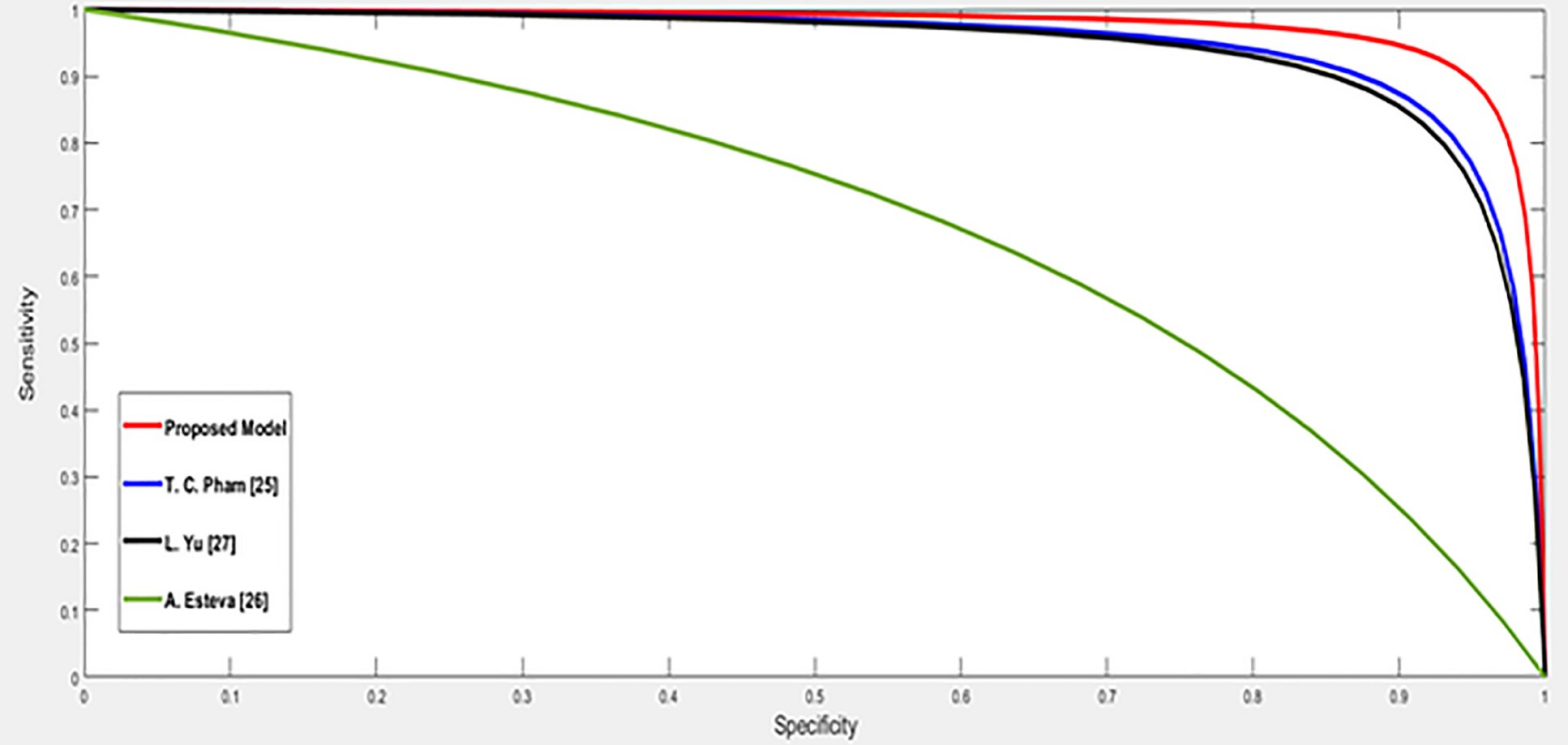
The ROC curves for the different methods with the ISIC dataset.

The ISIC dataset is used in the last comparative group. In these existing methods [24, 25, 26], the input images are enhanced and segmented. The obtained results for the proposed method and the existing classification methods [25, 26, 27] are shown in Table 4 and displayed in a visual form in Fig 10. The ROC curves which represent the relation between sensitivity and specificity for the proposed and the existing methods are plotted and displayed in Fig 11.

**Table 4:** Comparative study for ISIC dataset.

	Preprocessing (Enhancement)	Segmentation	Image Type	Classification Method	Accuracy (%)	Sensitivity (%)	Specificity (%)
Esteva et al. [26]	Yes	Yes	RGB	SVM	72.1	-	-
Yu et al. [27]	Yes	Yes	Gray-scale	CNN	85.5	50.7	94.1
Pham et al. [25]	Yes	Yes	RGB	SVM	89	55.6	97.1
Proposed Method	No	Yes	RGB	DCNN	95.91	88.47	93.00

For DermIS- DermQuest dataset; Amelard et al. [14] gained 87.38% for accuracy when using SVM for classification after enhancing the skin images and extracting the region of interest by segmentation. This method works with the RGB color images. In [15], Karabulut, and Ibrikci reduced the noise by enhancing the images and then applied the segmentation process to get the ROI regions. They used the CNN and the SVM for classification where the achieved accuracy was 71.4%. This method works with grayscale images. Almaraz et al. [16] utilized similar steps where the achieved accuracy was 75.1%. On the other side, the proposed method achieved an accuracy of 96.86% without any kind of enhancement.

All of the existing methods [17, 18, 22, 23, and 24] used the MED-NODE dataset. In these methods, two pre-processing steps were applied and kept the input images in R, G, and B color space except the method in [24] in which the input image converted to grayscale. The input RGB color images are enhanced and then the segmentation process was performed. Different classification methods such as probabilistic neural network, SVM, majority vote, and DLNN were used. The highest accuracy was 92.89% achieved by the method [24]. Our proposed method achieved an accuracy 95.91% with the segmentation step without any enhancement of the input image in the R, G, and B space.

For the ISIC dataset, Esteva et al. [26] achieved an accuracy 72.1% by using the SVM classifier with enhanced color skin images and a segmentation process to extract the region of interest. Yu et al. [27] enhanced the image to reduce the noise and segmented the input image to extract the region of interest where Yu and his co-authors converted the input images to grayscale. The achieved accuracy of their model was 85.5% by using the CNN for classification. Pham et al. [25] enhanced the input images and extracted the region of interest. Then, used the SVM to classify the pre-processed color images where their model achieved the accuracy 87.2%. On the other side, our proposed model achieved high accuracy rate 95.91%.

The performed comparisons clearly show that the performance of the proposed method outperforming all existing skin lesions classification methods.

## Conclusion

To build a new deep neural network with high performance, a huge number of labeled images is needed. The proposed method applies the transfer learning in three different ways to pre-trained architecture. The classification layer of AlexNet is replaced by softmax layer to classify the skin lesion into two or three classes. Based on its flexible architecture, it can be used to classify skin lesions into more classes. The weights are fine-tuned and the datasets are augmented by different rotation angles to overcome the problem of overfitting. The performance of the proposed method is tested using three datasets, DermIS- DermQuest, MED-NODE, and ISIC using GPU. The average accuracy, average sensitivity, average specificity, and average precision for the proposed method with the DermIS- DermQuest are 96.86%, 96.90%, 96.90%, and 96.92% respectively. For the MED-NODE dataset the average values of the performance measures are 97.70%, 97.34%, 97.34%, and 97.93%, respectively. The average performance measures with the ISIC dataset are 95.91%, 88.47%, 93.00%, and 92.34%. The experimental results show that the proposed skin lesion classification method outperforms several state-of-the-art classification methods.

## Supporting information

S1 Table This table contains link to used datasets. (DOCX)

## References

[1] American Cancer Society: Cancer facts and figures 2018. Available from: https://www.cancer.org/content/dam/cancer-org/research/cancer-facts-and-statistics/annual-cancer-facts-and-figures/2018/cancer-facts-and-figures-2018.pdf, Cited 2 Aug 2018.

[2] Codella N., Nguyen Q., Pankanti S., Gutman D., Helba B., Halpern A., Smith J., “Deep learning ensembles for melanoma recognition in dermoscopy images”, IBM Journal of Research and Development. Vol.61, no.4/5, pp. 5:1–5:15, 2017.

[3] Ballerini L., Fisher R., Aldridge B., and Rees J., “A color and texture based hierarchical K-NN approach to the classification of non-melanoma skin lesions” in Color Medical Image Analysis, Lecture Notes in Computational Vision and Biomechanics Volume 6, Editors: (Emre Celebi M. and Gerald Schaefer), Springer, pp. 63–86, 2013.

[4] Codella N., Cai J., Abedini M., Garnavi R., Hapern A., and Smith J., “Deep learning, sparse coding, and SVM for melanoma recognition in dermoscopy images”, Machine Learning in Medical Imaging. Lecture Notes in Computer Science, Springer, vol. 9352, pp. 118–126, 2015.

[5] Mishra N., and Celebi M., “An overview of melanoma detection in dermoscopy images using image processing and machine learning”, 2016. Available from: arXiv:1601.07843, Cited 2 Aug 2018.

[6] Apalla Z., Nashan D., Weller R., Castellsagué X., “Skin Cancer: Epidemiology, Disease Burden, Pathophysiology, Diagnosis, and Therapeutic Approaches” Dermatol Ther (Heidelb), Vol. 7, pp. 5–19, 2017. 28150105 10.1007/s13555-016-0165-y PMC5289116

[7] Jerant A., JOHNSON J., SHERIDAN C., and CAFFREY T., “Early detection and treatment of skin cancer,” Amer. Family Phys., vol. 62, no. 2, pp. 357–386, 2000. 10929700

[8] Binder M., Schwarz M., Winkler A., Steiner A., Kaider A., Wolff K., et al “Epiluminescence microscopy. A useful tool for the diagnosis of pigmented skin lesions for formally trained Dermtologists,” Arch. Dermtol., vol. 131, no. 3, pp. 286–291, 1995. 10.1001/archderm.131.3.286 7887657

[9] Siegel RL., Miller KD., and Jemal A., “Cancer statistics, 2018,” CA. Cancer J. Clin., vol. 68, no.1, pp.7–30, 2018 10.3322/caac.21442 29313949

[10] Barata C., Celebi M., and Marques J., "A Survey of Feature Extraction in Dermoscopy Image Analysis of Skin Cancer," in IEEE Journal of Biomedical and Health Informatics, vol. 23, no. 3, pp. 1096–1109, 2018 10.1109/JBHI.2018.2845939 29994234

[11] Celebi ME, Kingravi HA, Uddin B, Iyatomi H, Aslandogan YA, Stoecker WV, et al“A methodological approach to the classification of dermoscopy images,” Comput. Med. Imag. Graph., vol. 31(6), pp. 362–373, 2007. 10.1016/j.compmedimag.2007.01.003 PMC3192405 17387001

[12] Tommasi T., La Torre E., Caputo B., “Melanoma recognition using representative and discriminative kernel classifiers” In Proceedings Computer Vision Approaches to Medical Image Analysis, Springer, vol. 4241, pp. 1–12, 2006.

[13] Pathan S., Prabhu k., Siddalingaswamy P., “A methodological approach to classify typical and atypical pigment network patterns for melanoma diagnosis”, Biomedical Signal Processing and Control, vol. 44 Pp. 25–37, 2018.

[14] Amelard R, Wong A, Clausi DA., “Extracting Morphological High-Level Intuitive Features (HLIF) for Enhancing Skin Lesion Classification”, Int. Conference of the IEEE Engineering in Medicine and Biology Society, pp.4458-4461, 2012. 10.1109/EMBC.2012.6346956 23366917

[15] Karabulut E., and Ibrikci T., “Texture analysis of Melanoma Images for Computer-aided Diagnosis”, Int. Conference on Intelligent Computing, Computer Science & Information Systems (ICCSIS 16), vol. 2, pp.26-29,2016.

[16] Almaraz J., Ponomaryov V., Gonzalez E., “Melanoma CADe based on ABCD Rule and Haralick Texture Features” in 9th Int. Kharkiv Symposium on Physics and Engineering of Microwaves, Millimeter and Submillimeter Waves (MSMW), IEEE, pp. 1–4, 2016. 10.1109/MSMW.2016.7537993

[17] Giotis I., Molders N., Land S., Biehl M., junkman M., and Petkov N., “MED-NODE: A computer-assisted melanoma diagnosis system using non-dermoscopic images”, Expert Systems with Applications, vol.42, no. 19, pp. 6578–6585, 2015.

[18] Jafari M., Samavi S., Karimi N., Soroushmehr S., Ward K., and Najarian K., “Automatic Detection of Melanoma Using Broad Extraction of Features from Digital Images”, in 38th Int. Con. of the IEEE Eng. in Medicine and Biology Society (EMBC), pp. 1357–1360, 2016. 10.1109/EMBC.2016.7590959 28268577

[19] Litjens G., Kooi T., Bejnordi B., Setio A., Ciompi F., Ghafoorian M., et al “A survey on deep learning in medical image analysis”, Medical Image Analysis, Vol. 42, pp. 60–88, 2017 10.1016/j.media.2017.07.005 28778026

[20] Schaefer G., Krawczyk B., Celebi M., and Iyatomi H., “An ensemble classification approach for melanoma diagnosis,” Memetic Comput., Springer, vol. 6, no. 4, pp. 233–240, 2014.

[21] Shin HC, Roth HR, Gao M, Lu L, Xu Z, Nogues I, et al, “Deep convolutional neural networks for computer aided detection: CNN architectures, dataset characteristics and transfer learning,” IEEE Trans. Med. Imaging, vol. 35, no. 5, pp. 1285–1298, 2016 10.1109/TMI.2016.2528162 26886976 PMC4890616

[22] Nasr-Esfahan E., Samavi S., Karimi N., Soroushmehr S., Jafari M., Ward K.et al. “Melanoma Detection by Analysis of Clinical Images Using Convolutional Neural Network”, Int. Conference of the IEEE Engineering in Medicine and Biology Society,137(2016), pp, 1373–1376. 10.1109/EMBC.2016.7590963 28268581

[23] Kostopoulos SA, Asvestas PA, Kalatzis IK, Sakellaropoulos GC, Sakkis TH, Cavouras DA, et al “Adaptable pattern recognition system for discriminating Melanocytic Nevi from Malignant Melanomas using plain photography images from different image databases”, International Journal of Medical Informatics, Vol. 105, pp. 1–10, 2017 10.1016/j.ijmedinf.2017.05.016 28750902

[24] Premaladha J., and Ravichandran K., “Novel Approaches for Diagnosing Melanoma Skin Lesions Through Supervised and Deep Learning Algorithms”, Journal of Medical Systems, Vol. 40, no. 96, pp. 1–12, 2016. 26872778 10.1007/s10916-016-0460-2

[25] Pham TC., Luong CM, Visani M., and Hoang VD, “Deep CNN and Data Augmentation for Skin Lesion Classification”, Intelligent Information and Database Systems, Lecture Notes in Computer Science, Springer, vol. 10752, pp. 573–582, 2018.

[26] Esteva A., Kuprel B., Novoa R., Ko J., Swetter S., Blau H., et al, “Dermtologist-level classification of skin cancer with deep neural networks”, Nature, vol. 542, pp. 115–118, 2017 10.1038/nature21056 28117445 PMC8382232

[27] Yu L., Chen H., Dou Q., Qin J. and Heng P., "Automated Melanoma Recognition in Dermoscopy Images via Very Deep Residual Networks," IEEE Transactions on Medical Imaging, vol. 36, no. 4, pp. 994–1004, 2017 10.1109/TMI.2016.2642839 28026754

[28] LeCun Y., Bengio Y., Hinton G., “Deep learning”, Nature, vol. 521, PP.436–444, 2015 10.1038/nature14539 26017442

[29] Tajbakhsh N., Shin J., Gurudu S., Hurst R., Kendall C., Gotway M., et al “Convolutional neural networks for medical image analysis: Full training or fine tuning?” IEEE Trans. Med. Imag., vol. 35, no. 5, pp. 1299–1312, 2016. 10.1109/TMI.2016.2535302 26978662

[30] Yang X., Zeng Z., Yeo S., Tan C., Tey H., and Su Y., “A Novel Multitask Deep Learning Model for Skin Lesion Segmentation and Classification”, CORR Preprint. Available from: arXiv:1703.01025, Cited 2 Aug 2018.

[31] Greenspan H., Ginneken B., and Summers R., “Guest editorial deep learning in medical imaging: Overview and future promise of an exciting new technique,” IEEE Trans. Med. Imag., vol. 35, no. 5, pp. 1153–1159, 2016.

[32] Wenpeng Y., Katharina K., Yu M., and Schutze H., “Comparative Study of CNN and RNN for Natural Language Processing”, CoRR, Preprint. Available from: arXiv:1702.01923v1, Cited 2 Aug 2018.

[33] Srinivas S., Sarvadevabhatla R., Mopuri K., Prabhu N., Kruthiventi S., Babu R.,"A taxonomy of Deep Convolutional Neural Nets for Computer Vision", Frontiers in Robotics and AI, Vol.2, 2016, Available from: arXiv:1601.06615, Cited 2 Aug 2018.

[34] Convolutional Neural Networks (CNNs / ConvNets), the Stanford CS class notes, Spring 2017 Assignments, Available: http://cs231n.github.io/convolutional-networks/, Accessed: 2 Aug 2018.

[35] Krizhevsky A., Sutskever I. and Hinton G., "ImageNet Classification with Deep Convolutional Neural Networks", In Proc. Neural Information Processing Systems (NIPS), vol. 1, pp.1097–1105, 2012.

[36] Deng J., Dong W., Socher R., Li LJ, Li K., and Fei-Fei L., “ImageNet: A large-scale hierarchical image database,” in Proc. IEEE Conf. Computer Vision and Pattern Recognition, pp. 248–255, 2009. 10.1109/CVPR.2009.5206848

[37] Gutman D., Codella N., Celebi E., Helba B., Marchetti M., Mishra N., et al., “Skin lesion analysis toward melanoma detection: A challenge at the international symposium on biomedical imaging (ISBI) 2016, hosted by the international skin imaging collaboration (ISIC)”, 2016, Available from: arXiv:1605.01397, Cited 2 Aug 2018

[38] Dermtology Information System, Available from: http://www.dermis.net, 2012, cited 2 Aug 2018.

[39] DermQuest, Available from: http://www.dermquest.com, 2012, cited 2 Aug 2018.

[40] Perez F., Vasconcelos C., Avila S., Valle E., “Data Augmentation for Skin Lesion Analysis”, Computer Assisted Robotic Endoscopy, Clinical Image-Based Procedures, and Skin Image Analysis, Lecture Notes in Computer Science, Springer,vol 11041, pp 303–311,2018.

[41] MATLAB Central Program or Color Image Segmentation–Athi Narayan S, K.S.R. College of Engineering, Erode, Tamil Nadu, India, Available from: https://www.mathworks.com/matlabcentral/fileexchange/25257-color-image-segmentation?focused=5191437&tab=function, cited 2 Aug 2018.

[42] Stojanovi M., Apostoloviü M., Stojanoviü D., Miloševiü Z., Toplaoviü A., Lakušiü V., et al, “Understanding sensitivity, specificity and predictive values”, Vojnosanit Pregl, vol. 71, no11, pp. 1062–1065,2014 25536811 10.2298/vsp1411062s

[43] Fawcett T., “An introduction to ROC analysis” Pattern Recognition Letter, Vol. 27, no. 8, p. 861–874, 2006.

