# Supplementary material for: Classification of skin lesions using transfer learning and augmentation with Alex-net
Source: PLoS One. 2019 May 21;14(5):e0217293. doi: 10.1371/journal.pone.0217293 (PMC6529006; doi:10.1371/journal.pone.0217293)
Supplement: S1 Table — (DOCX) [file pone.0217293.s001.docx]

| Dataset name | Link |
| --- | --- |
| ISIC 2017 | <https://challenge.kitware.com/#challenge/n/ISIC_2017%3A_Skin_Lesion_Analysis_Towards_Melanoma_Detection> |
| Med-Node | <http://www.cs.rug.nl/~imaging/databases/melanoma_naevi/> |
| Demis | <https://zope.dermis.net/e04info/index_eng.html> |
| DermQuest | <https://www.derm101.com/dermquest/> |

We were used DermIS and DermQuest dataset as discussed in [14] to be that same strategy for comparison.

1. R Amelard, A. Wong, and D. A. Clausi, “Extracting Morphological High-Level Intuitive Features (HLIF) for Enhancing Skin Lesion Classification”, Int. Conference of the IEEE Engineering in Medicine and Biology Society, pp.4458-4461, 2012.
